# An International External Validation and Revision of the PsyMetRiC Cardiometabolic Risk Prediction Algorithm for Young People with Psychosis

**DOI:** 10.1192/j.eurpsy.2022.1741

**Published:** 2022-09-01

**Authors:** B. Perry, F. Vandenberghe, E.F. Osimo, C. Grosu, M. Piras, P. Jones, P. Mallikarjun, J. Stochl, R. Upthegrove, G. Khandaker, C. Eap

**Affiliations:** 1 University of Cambridge, Dept Of Psychiatry, Cambridge, United Kingdom; 2 Lausanne University Hospital, Chuv, Lausanne, Switzerland; 3 Imperial College London, Institute Of Clinical Sciences, London, United Kingdom; 4 University of Birmingham, Institute Of Clinical Sciences, Birmingham, United Kingdom

**Keywords:** Psychosis, risk prediction, young adults, cardiometabolic

## Abstract

**Introduction:**

The comorbidity between cardiometabolic and psychotic disorders develops early. This is a crucial window of opportunity to reduce excess morbidity and mortality. Recently, a cardiometabolic risk prediction algorithm for young people with psychosis, the psychosis metabolic risk calculator (PsyMetRiC) was developed and externally validated in the UK. However, its international transportability is unknown.

**Objectives:**

We performed the first international validation study of PsyMetRiC in Lausanne, Switzerland, and examined whether additional variables (clinical and/or genetic) may improve the predictive performance of the algorithm

**Methods:**

We included people aged 16-35y with psychosis from the PsyMetab cohort, who did not have MetS at baseline, and who had 1-6y follow-up data. The PsyMetRiC partial (age, sex, ethnicity, body mass index, smoking status, and prescription of a metabolically-active antipsychotic) and full (also including high-density lipoprotein and triglycerides) algorithms were applied. Predictive performance was assessed using measures of discrimination (C-statistic) and calibration (calibration plots). Recalibration steps included refitting the intercept and/or slope if necessary. Additional variables (e.g. speed of weight gain, polygenic risk scores) were added to the model and predictive performance was reassessed.

**Results:**

We included 545 participants. The discrimination performance of both PsyMetRiC algorithms was good (C>0.75), and calibration plots showed good agreement between observed and predicted risk. Additional analyses to be conducted.

**Conclusions:**

PsyMetRiC is likely to be generalizable for use in Switzerland, suggesting that PsyMetRiC may also be suitable for use in other European populations. While additional international validations are required, these findings are an encouraging step toward an international cardiometabolic risk prediction algorithm for young people with psychosis.

**Disclosure:**

No significant relationships.

